# A better integration of health and economic impact assessments of climate change

**DOI:** 10.1088/1748-9326/ad29a9

**Published:** 2024-02-23

**Authors:** Anton Orlov, Jessie Schleypen, Kristin Aunan, Jana Sillmann, Antonio Gasparrini, Malcolm N Mistry

**Affiliations:** 1 CICERO Center for International Climate Research, Oslo, Norway; 2 Climate Analytics, Berlin, Germany; 3 Sustainability and Climate Risks, University of Hamburg, Hamburg, Germany; 4 Environment & Health Modelling (EHM) Lab, Department of Public Health Environments and Society, London School of Hygiene & Tropical Medicine, London, United Kingdom; 5 Department of Economics, Ca’ Foscari University of Venice, Venice, Italy

**Keywords:** health, economy, climate change, model integration

## Abstract

Climate change could lead to high economic burden for individuals (i.e. low income and high prices). While economic conditions are important determinants of climate change vulnerability, environmental epidemiological studies focus primarily on the direct impact of temperature on morbidity and mortality without accounting for climate-induced impacts on the economy. More integrated approaches are needed to provide comprehensive assessments of climate-induced direct and indirect impacts on health. This paper provides some perspectives on how epidemiological and economic impact assessments could be better integrated. We argue that accounting for the economic repercussions of climate change on people’s health and, vice versa, the consequences of health effects on the economy could provide more realistic scenario projections and could be more useful for adaptation policy.

## 1 Introduction

The link between temperature and mortality is among the most well documented. Growing empirical evidence has documented a causal and continuous relationship between non-optimal temperature and mortality underpinning the existence of location-specific optimal temperature levels (i.e. by a U-shaped (convex) temperature-mortality relationship implying minimum mortality temperatures). Hot temperatures are shown to worsen cardiovascular diseases, which are a major cause of death globally, but also other common diseases, such as respiratory diseases and chronic kidney diseases (Ebi *et al* 2021). Such empirical studies are typically based on time series regression, and while environmental epidemiological (EE) models usually differ with respect to the functional form and the set of explanatory variables, the majority of them account for a non-linear lagged relationship between temperature and mortality (Gasparrini *et al* 2017, Vicedo-Cabrera *et al* 2018). In addition to temperature, some models also include vulnerability covariates, such as income that is typically measured by the gross domestic product (GDP) per capita (Gasparrini *et al* 2017). The estimated observation-based temperature-mortality exposure-response functions can then be used to project excess mortality under different greenhouse gas emissions scenarios into the future. Recent studies have found that global warming is projected to increase the risk of temperature-related morbidity and mortality, particularly in low-latitude and low-income countries where many people are exposed to heat stress (Vicedo-Cabrera *et al* 2018).

However, a majority of such projections are based on fixed (present-day) income levels (Gasparrini *et al* 2017). While some advanced EE models include various demographic characteristics, such as age and gender, the representation of economic vulnerability factors remains oversimplified. A few studies rooted in econometrics have also projected the burden of future changes in temperature on mortality, by utilising future GDP projections derived from the Shared Socioeconomic Pathways (Carleton *et al* 2022). However, the climate-induced impacts on income and inequality are not accounted for in the study by Carleton *et al* (2022). For instance, the authors demonstrate that the estimates not accounting for adaptation, which is likely to increase as a result of economic growth, could substantially over-state the mortality effects. In this regard, neglecting the dynamics of socio-economic factors could lead to biased results.

**Figure 1:**
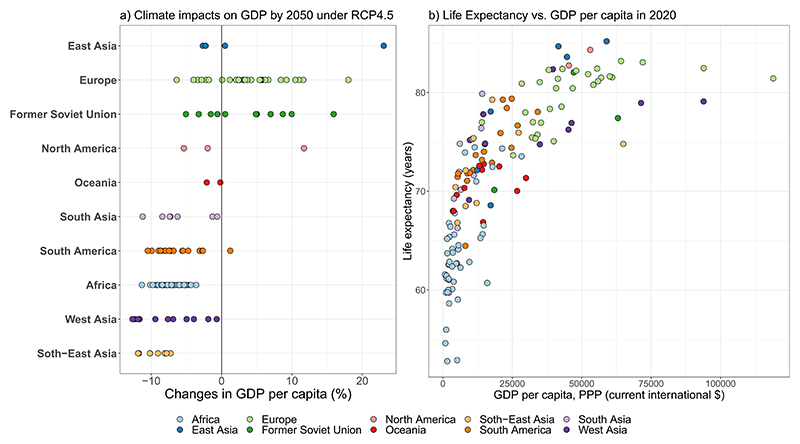
(a) Temperature-induced impacts on economic growth by 2050 relative to 2010 under RCP4.5, which are estimated using the econometric model from Burke *et al* (2018) and Phase 5 of the Coupled Model Intercomparison Project data. The circles show countries within a region. (b) Life expectancy vs. GDP per capita. The circles show countries within a region. Source: data on life expectancy is from United Nations Population Division (the 2022 Revision of World Population Prospects) and data on GDP per capita is from World Bank (Word Development Indicators database).

At the same time, existing empirical studies have shown that, apart from the direct climate-related impacts on death and disease, climate change could also lead to a substantial reduction in economic productivity in low-latitude countries (Pretis *et al* 2018, Diffenbaugh and Burke 2019). In some low-latitude countries, climate-induced reductions in real GDP could account for more than 10% by the middle of the century (figure 1(a)). For low-income groups within a country, a relative reduction in income could surpass a reduction in GDP. In contrast, some high-latitude countries could experience a higher economic growth. In this context, accounting for the climate-induced economic impacts in scenario projections would enable assessment of inequality in terms of the health impacts (i.e. climate justice). The empirical relationship between temperature (or both temperature and precipitation) and economic growth implemented in such studies is generally estimated using econometric models (i.e. panel regression models). Typically, econometric models show a non-linear inverted U-shaped relationship between temperature and economic growth, although the estimated relationship can remain sensitive to the choice of the econometric specification (Newell *et al* 2021). Beyond the econometric approaches applied for assessing economic impacts of climate change, the economic impacts of climate change are also evaluated using economic process-based (PB) models, such as partial equilibrium and computable general equilibrium models, and agent-based models. PB models are based on macro- and micro-economic principles and use inputs from climate-impact models and/or exposure-response functions, which estimate the bio-physical effects (i.e. crop yields, labour and capital productivity) of climate change. PB-based economic studies have also found substantial costs of climate change under high warming scenarios (van der Wijst *et al* 2023). Economic wealth is an important vulnerability factor, and the economy-health nexus is often complex and country-specific. The remainder of this paper addresses the economy-health association and provides some future research perspectives on how to better integrate epidemiological and economic impact assessments.

## 2 Economy-health nexus

Socioeconomic factors are known to affect the interaction between climate and health (Cromar *et al* 2021), with several theoretical and empirical studies showing a significant bidirectional relationship between income and health (Kunze 2014, He and Li 2020, Miladinov 2020). On the one hand, economic growth leads to a higher income, which implies better access to private health services, though income inequality may persist (Prados de la Escosura 2023). Economic growth can also increase fiscal capacity to fund the public health sector, and a higher private income allows affordability of private health services. Richer societies have better living conditions enabling people to avoid thermal discomfort (e.g. air conditioning and more space) and can afford healthier diets. Also, in less economically developed countries, a larger share of the population is involved in the primary sector (i.e. production of raw materials), which can be very physically demanding if not mechanised. In contrast, in economically developed countries, relatively more people are involved in the secondary (manufacturing) and tertiary sector (services), which typically rely on less strenuous work than in the primary sector. Furthermore, economic growth is associated with mechanisation deployment, which can reduce work intensity and vulnerability to heat (Orlov *et al* 2020). Thus, countries with higher incomes tend to have a longer life expectancy (figure 1(b)). Alongside with income, inflation determines the cost of living and welfare of individuals. High food prices and low income can worsen affordability of food and healthy diets, thereby increasing the risk to health for the poorer part of the population, which could particularly affect the health of children, due to undernutrition (Lee *et al* 2016, Kidane and Woldemichael 2020). Furthermore, lines of evidence show a potential impact of suboptimal diets on non-communicable diseases, which induce mortality and morbidity (Afshin *et al* 2019, Blakely *et al* 2020).

On the other hand, better health services can stimulate economic growth through an increase in labour capacity (i.e. labour force) (Gürler and Özsoy 2019). While a higher life expectancy could provide increased labour force, it does not necessarily increase per capita economic growth (Acemoglu and Johnson 2007). A higher life expectancy also implies a higher share of elderly people. As older adults are more susceptible to adverse effects of heat, this can increase the overall impact of climate change and the need for health services. Overall, the economy-health nexus is complex and country- and individual-specific (Ruhm 2000, Stafoggia *et al* 2021) and a diverse set of indicators of economic conditions are important to include in analyses as they can affect health through different mechanisms. However, research on the economic consequences of climate change has largely focused on how climate-induced changes in morbidity and mortality will affect the labour market and economic growth (Bosello *et al* 2006, García-León *et al* 2021). The economic repercussions of climate change on health have yet, to the best of our knowledge, not been integrated and quantified in health impact assessments. Economic and epidemiological impact assessments related to climate change thus remain largely non-integrated. This implies that previous studies on the links between climate change and health might not provide comprehensive and realistic estimates, as correctly acknowledged in their limitations (e.g. Gasparrini *et al* 2017, Carleton *et al* 2022).

## 3 Outlook

**Figure 2:**
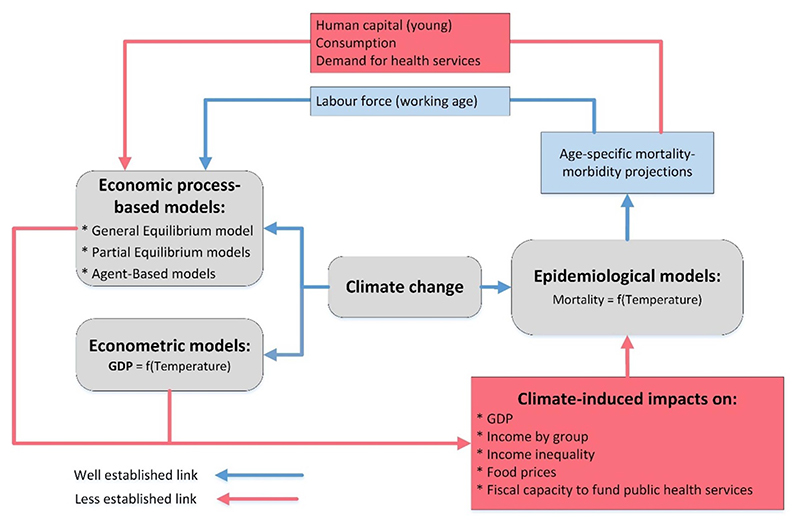
Climate-economy-health modelling interface.

Developing a comprehensive assessment of climate-related impacts on health needs a better collaboration across disciplines, which should facilitate integration and modularity of different modelling approaches. Conscious of the fact that climate-induced impacts on economic vulnerability factors (i.e. income and prices) are yet to be conceptualised and implemented in the state-of-the-art EE models, we propose some concrete steps for model integration towards a more comprehensive assessments of climate-induced impacts on health. Specifically, we discuss a potential integration with econometric and economic PB models, which are two main modelling approaches widely used for assessing the economic cost of climate change (figure 2).

### 3.1 Integration with econometric models

One of the advantages of econometric models, is their comprehensiveness in terms of climate impacts. Econometric models can capture all possible temperature- and precipitation-related impacts on economic growth without explicitly modelling the transmission channels. Climate-induced impacts on the economic growth estimated by econometric models can be incorporated into epidemiological models to project future health impacts. For instance, projections for GDP including climate impacts derived from econometric models can be included in the EE models instead of assuming fixed values. Also, since new economic data is collected and generated, sub-national GDP projections can be implemented in EE models. Importantly, both EE and econometric modelling should move beyond using GDP as a proxy variable for economic vulnerability. GDP is an aggregated economic index that hides important income heterogeneity across different household groups. EE models could potentially include some indexes of inequality alongside with GDP, such as the Gini index or Human Development Index (Kummu *et al* 2018).

### 3.2 Integration with economic PB models

Similarly, PB models can alternatively provide input data to EE projection models. Compared to econometric models, PB models are often less comprehensive in terms of climate impacts and have a weaker empirical foundation. However, PB models can provide a richer set of economic variables, and they could be more suitable for modelling adaptation. For example, some multi-sectoral economic models can quantify the sector-specific price and income responses (e.g. food prices), and employment effects (Carbone *et al* 2022). Specifically, food prices could potentially be used for quantifying the indirect impact of climate change on morbidity and mortality. Also, large scale macro-economic models can be linked with microeconomic simulations based on household expenditure surveys, which allow to quantify the income impact for different income groups. Multi-sectoral economic models can also simulate labour mobility across sectors within an economy, which could also be useful input data in occupational health assessments. In this regard, general equilibrium economic models can provide estimates on fiscal capacity to fund public health services, which in turn could be integrated into EE modelling framework.

However, to fully utilize the detailed output from PB economic models, EE models need to be advanced further. Specifically, the regional and sub-regional associations between income and food prices, and health need to be estimated and consistently integrated into EE models. Economic vulnerability in EE models should be represented by household-specific income levels and occupation instead of national GDP. Reversely, output from EE models could be better utilised in economic models, which primarily use mortality projections to quantify the impact on labour markets. Specifically, age-specific mortality and morbidity projections can be used to assess the impacts on consumption, demand for health services, and human capital (i.e. ability of young people to contribute to economic productivity) (figure 2).

Overall, a successful model integration requires cross-disciplinary collaboration and development in both EE and economic modelling. Collection of new epidemiological and socio-economic data, in terms of outcomes, geographical coverage, and spatial and temporal resolution will further facilitate the model integration. At present, mortality and socio-economic data are missing for many countries that are exposed to heat stress (e.g. African countries). A better integration of EE and economic models would allow not only improving the projections of climate-induced impacts on health but would also help design adaptation policies. We propose that integration of health and economic impact assessments could be an alternative approach to the traditional and disputed method of using the value of a statistical life, which only expresses mortality impacts in monetary terms.

## Data Availability

No new data were created or analysed in this study.

## References

[R1] Acemoglu D, Johnson S (2007). Disease and development: the effect of life expectancy on economic growth. J Polit Econ.

[R2] Afshin A (2019). Health effects of dietary risks in 195 countries, 1990–2017: a systematic analysis for the Global Burden of Disease Study 2017. Lancet.

[R3] Blakely T, Cleghorn C, Mizdrak A, Waterlander W, Nghiem N, Swinburn B, Wilson N, Mhurchu CN (2020). The effect of food taxes and subsidies on population health and health costs: a modelling study. Lancet Publ Health.

[R4] Bosello F, Roson R, Tol RSJ (2006). Economy-wide estimates of the implications of climate change: human health. Ecol Econ.

[R5] Burke M, Davis WM, Diffenbaugh NS (2018). Large potential reduction in economic damages under UN mitigation targets. Nature.

[R6] Carbone JC, Bui LTM, Fullerton D, Paltsev S, Sue Wing I (2022). When and how to use economy-wide models for environmental policy analysis. Annu Rev Resour Econ.

[R7] Carleton T (2022). Valuing the global mortality consequences of climate change accounting for adaptation costs and benefits. Q J Econ.

[R8] Cromar K, Howard P, Vásquez VN, Anthoff D (2021). Health impacts of climate change as contained in economic models estimating the social cost of carbon dioxide. GeoHealth.

[R9] Diffenbaugh NS, Burke M (2019). Global warming has increased global economic inequality. Proc Natl Acad Sci USA.

[R10] Ebi KL (2021). Hot weather and heat extremes: health risks. Lancet.

[R11] García-León D, Casanueva A, Standardi G, Burgstall A, Flouris AD, Nybo L (2021). Current and projected regional economic impacts of heatwaves in Europe. Nat Commun.

[R12] Gasparrini A (2017). Projections of temperature-related excess mortality under climate change scenarios. Lancet Planet Health.

[R13] Gürler M, Özsoy Ö (2019). Exploring the relationship between life expectancy at birth and economic growth in 56 developing countries. J Glob Health Rep.

[R14] He L, Li N (2020). The linkages between life expectancy and economic growth: some new evidence. Empir Econ.

[R15] Kidane D, Woldemichael A (2020). Does inflation kill? Exposure to food inflation and child mortality. Food Policy.

[R16] Kummu M, Taka M, Guillaume JHA (2018). Gridded global datasets for gross domestic product and human development index over 1990–2015. Sci Data.

[R17] Kunze L (2014). Life expectancy and economic growth. J Macroecon.

[R18] Lee H-H, Lee SA, Lim J-Y, Park C-Y (2016). Effects of food price inflation on infant and child mortality in developing countries. Eur J Health Econ HEPAC Health Econ Prev Care.

[R19] Miladinov G (2020). Socioeconomic development and life expectancy relationship: evidence from the EU accession candidate countries. Genus.

[R20] Newell RG, Prest BC, Sexton SE (2021). The GDP-temperature relationship: implications for climate change damages. J Environ Econ Manage.

[R21] Orlov A, Sillmann J, Aunan K, Kjellstrom T, Aaheim A (2020). Economic costs of heat-induced reductions in worker productivity due to global warming. Glob Environ Change.

[R22] Prados de la Escosura L (2023). Health, income, and the Preston curve: a long view. Econ Hum Biol.

[R23] Pretis F, Schwarz M, Tang K, Haustein K, Allen MR (2018). Uncertain impacts on economic growth when stabilizing global temperatures at 1.5 °C or 2 °C warming. Phil Trans A.

[R24] Ruhm CJ (2000). Are recessions good for your health?. Q J Econ.

[R25] Stafoggia M (2021). Effects of air temperature on cardiopulmonary mortality and morbidity in Europe (Zenodo).

[R26] van der Wijst K-I (2023). New damage curves and multimodel analysis suggest lower optimal temperature. Nat Clim Change.

[R27] Vicedo-Cabrera AM (2018). Temperature-related mortality impacts under and beyond Paris Agreement climate change scenarios. Clim Change.

